# Machine Learning Meets Compressed Sensing in Vibration-Based Monitoring

**DOI:** 10.3390/s22062229

**Published:** 2022-03-14

**Authors:** Federica Zonzini, Antonio Carbone, Francesca Romano, Matteo Zauli, Luca De Marchi

**Affiliations:** 1 Advanced Research Center on Electronic Systems “Ercole De Castro” (ARCES), University of Bologna, 40136 Bologna, Italy; federica.zonzini@unibo.it (F.Z.); antoniocarbone095@gmail.com (A.C.); frangaromano@gmail.com (F.R.); matteo.zauli7@unibo.it (M.Z.); 2 Department of Electrical, Electronic and Information Engineering (DEI), University of Bologna, 40136 Bologna, Italy

**Keywords:** artificial intelligence, MEMS accelerometers, model-assisted takeness-based compressed sensing, operational modal analysis, structural health monitoring

## Abstract

Artificial Intelligence applied to Structural Health Monitoring (SHM) has provided considerable advantages in the accuracy and quality of the estimated structural integrity. Nevertheless, several challenges still need to be tackled in the SHM field, which extended the monitoring process beyond the mere data analytics and structural assessment task. Besides, one of the open problems in the field relates to the communication layer of the sensor networks since the continuous collection of long time series from multiple sensing units rapidly consumes the available memory resources, and requires complicated protocol to avoid network congestion. In this scenario, the present work presents a comprehensive framework for vibration-based diagnostics, in which data compression techniques are firstly introduced as a means to shrink the dimension of the data to be managed through the system. Then, neural network models solving binary classification problems were implemented for the sake of damage detection, also encompassing the influence of environmental factors in the evaluation of the structural status. Moreover, the potential degradation induced by the usage of low cost sensors on the adopted framework was evaluated: Additional analyses were performed in which experimental data were corrupted with the noise characterizing MEMS sensors. The proposed solutions were tested with experimental data from the Z24 bridge use case, proving that the amalgam of data compression, optimized (i.e., low complexity) machine learning architectures and environmental information allows to attain high classification scores, i.e., accuracy and precision greater than 96% and 95%, respectively.

## 1. Introduction

Damage detection has a pivotal role in Structural Health Monitoring (SHM) systems as a fundamental means to implement on-condition maintenance. In particular, many novel damage detection procedures are gaining momentum thanks to the recent developments in the Machine Learning (ML) field [1,2,3,4,5]. Indeed, to cope with these continuously evolving requirements, novel Artificial Intelligence (AI) tools have been proposed in the recent literature, which were fostered by the parallel technological advancements in the processing power promoted by the information engineering community. In seminal works, the adoption of graph convolutional networks, in which the problem of feature extraction and classification is mapped in the graph domain rather than resorting to the standard time/frequency representation, has shown the benefit of learning data patterns in a more flexible and self–adaptive way. For example, in [6,7], graph models were applied for crack detection and localization in the framework of vibration diagnostic, showing outstanding performances. Capsule neural networks have also demonstrated great potential to tackle SHM issues. These networks, which are intrinsically based on convolutional operations, are unique in that, thanks to the novel concept of capsule unit and routing by agreement, they can overcome the main limitations of conventional Convolutional Neural Networks, such as shift and rotation invariance and the presence of mandatory pooling layers, while preserving spatial relationships among the learned features. As a representative use case, capsule neural networks were applied for source localization purposes [8], showing greater generalization performances with respect to standard convolutional models.

Another emerging approach, Spiking Neural Networks (SNN), was recently adopted for damage assessment purposes. SNNs are peculiar in that they try to tackle the problem of structural inference by means of a more realistic mathematical representations of the human brain, which specifically mimics biological spike-based event-driven processes to communicate between neurons [9] (a research perspective which is also known as neuromorphic computing). The main advantage of these models is that, when implemented on custom hardware, they are more power efficient than standard AI approaches. Inspired by this idea, researchers in [10] have proven that SNNs can be very performative for vibration-based assessment, and suitable to be embedded on resource-constrained device, with considerable power saving for the underlying electronics.

It is also worth mentioning the continual learning paradigm [11], in which the trained diagnostic models are continuously updated, over time and in real time, without the bottlneck of performing long data collection phases to train the networks at the beginning of the SHM system lifecycle. In this way, comparatively tinier models could be designed, which are most suited for near-sensor integration, hence reducing the latency and the cost of the monitoring process.

In this context, anomalies are identified by feeding ML algorithms with damage sensitive features which are deemed to be representative of the structure under inspection [12]. In particular, the integrity assessment of structures in the dynamic regime usually relies on the extraction of vibration parameters, the so-called modal parameters, which comprise natural frequencies, damping ratio and mode shapes [13]. When the excitation signal is unknown, a condition which is typical for the majority of the civil and industrial plants, modal features are extracted by resorting to operational modal analysis (OMA) techniques [14].

The modal identification process implies long time series are to be collected, stored and processed for each sensing device. If the structure under inspection has large dimensions and complex geometries, which demand the deployment of very dense sensor networks basing on low-cost sensors [15], the risk of having unacceptable data flooding and network congestion is high. Prompted by these issues, data compression techniques were investigated as viable solutions to alleviate the communication and memory burden caused by such large datasets. Spanning from methodologies based on compressed sensing (CS) [16] up to AI-driven alternatives [17], a wide range of solutions have been developed in the last few years to fulfill this task.

The performance of compression approaches is usually evaluated by computing the mean square error between the recovered and the originally acquired signals [16,18], or by analysing the degradation in the modal parameter estimation [19,20], while only minor attention (to the best of these authors’ knowledge) was paid to assess how compression affects the damage classification performance. Indeed, the list of works dealing with the combination of data compression and ML is relatively short and includes very general application scenarios. For example, authors in [21] proposed a compressed sensing and online extreme learning autoencoder for anomaly detection in Internet of Things frameworks, while the problem of efficient data management and anomaly/attack identification in wireless sensor networks was discussed in [22]. A combination of random projection serving the task of data reduction while preserving anomalous data in image processing can instead be found in [23]. However, none of these works tackles the specificity of structural health monitoring, such as the necessity to estimate modal parameters.

As far as classification is concerned, ML and deep learning architectures targeting the identification of structural damages were extensively investigated (see [3,24]). Nevertheless, as anticipated, an important challenge is the embedding of inference algorithms on the smart sensing nodes at the extreme edge [25], i.e., the adoption of a ‘sensor-near’ monitoring paradigm where information is partly elaborated on local sensors, in strict proximity where it is actually sensed. This trend translates into the design of tiny ML architectures, which correspond to small-size, low-complexity and minimal power consumption AI applications [26] compliant with hardware-oriented solutions. Hence, stringent limits are imposed concerning the complexity of the classification models to be deployed, given the constrained computational resources available on the sensor nodes.

Furthermore, condition monitoring data are typically affected by environmental and operational parameters (EOPs), whose effect on modal parameters can be even more pronounced than the one due to pure structural degradation [27]. Thus, EOPs need to be properly modeled and taken into consideration to avoid false alarms. Dynamic regression analysis and principal component analysis are just a few of the reference approaches already investigated in the field [28]. All these methodologies aim at finding non-linear dependencies between the measured environmental factors and the identified structural parameters, which are then compensated by means of standard fitting models. Alternatively, more sophisticated methodologies to the ones based on standard eigen analysis have recently started to be considered, such as the one offered by singular spectrum analysis that is specifically designed to identify and extract oscillatory components from time series [29] while filtering out the presence of unwanted, aperiodic noise trends. This algorithm is implemented via the cascade of an embedding (forward) and grouping (inverse) operation with an intermediate step of spectral decomposition, in which each individual time series (and not a whole batch of data as required by classical principal component) is decomposed into its individual components, thus allowing for a better removal of inherent noise and seasonal drifts without affecting the quality of the modal features [30].

In such scenario, the primary aim of classification networks is to determine whether degrading phenomena are occurring or not and to signal alerts in a timely manner, a task which is usually referred to as One Class Classification (OCC). The objective of OCC is, therefore, to find which specific class a given input object belongs to by selecting either the *target* (i.e., ‘normal’) or *outlier* (i.e., ‘anomalous’) class. OCC solutions based on standard neural network (NN) models were shown to achieve good classification scores in numerous application scenarios, such as the monitoring of industrial plants (e.g., mechanical rotors [31], wind farms [32,33]) and avionics or automotive structures [34,35]. In the civil engineering domain, a two-stage OCC Neural Network (OCCNN) was recently proposed [36] and validated on the dataset related to the Z24 bridge [37]. Indeed, this infrastructure has become a reference test bench for ML validation purposes [38,39]. In fact, the performances of the approach adopted in [36] are very promising, with a reported accuracy of 96% and a precision of 98%. However, the quality of the results provided by OCCNN is strongly influenced by the training set point distribution, whilst completely neglecting the dependence of the identified structural features from EOPs, a procedure which, in turn, does not allow to decouple the actual effect of structural degradation on the one due to operational variability. This means that even if a long data collection phase, usually performed on a yearly or at least seasonal scale, has to be conducted to create a set of baseline values comprehensive of all the possible structural-to-EOP dependencies, the retrieved information might yet be insufficient when these environmental changes are not properly compensated. Furthermore, the framework proposed by the authors in [36] does not include the degrading effect of lossy transmissions to central processing units, is *high-computationally demanding* and does not take into consideration the practical limitations of instrumentation non-idealities, such as the effect of intrinsic noise density native in low-cost devices; therefore, it is not suited to cope with the limited computational and storage resources available for embedded systems.

### Contribution

In this work, an OCCNN-based damage detection procedure for dense accelerometer networks is proposed and tested against the possible limitation of commercial off-the-shelf MEMS sensors. Such a procedure is compatible with the deployment of large sensor networks and embedded processing solutions. More specifically, the main contributions can be listed as follows:

- *Input data.* It is investigated how CS methodologies, which reduce the probability of network congestion, may affect the classification process. Temperature values are provided as additional input data for the NN machine to inherently model the dependency of modal features on environmental factors.
- *Knowledge distillation.* A reduction in the complexity of the NN models is performed by shrinking the number of neurons in the hidden layers, without affecting the classification accuracy with respect to more redundant configurations.
- *MEMS noise density.* Acceleration waveforms are corrupted with the intrinsic noise density characterizing MEMS-based sensors, which are the most widely adopted sensing technology in this kind of application and, thus, need to be properly handled in view of real installations. Hence, the robustness of the classification process under this technological limitation is evaluated.

The paper is organized as follows. The complete processing flow, from the data compression/decompression stage up to the classification process, is thoroughly illustrated in Section 2. Section 3 concerns the description of the experimental validation phase, which uses the the Z24 dataset as a reference application scenario. Results are presented in Section 4, discussing how the novel approaches explored in the work might increase the overall performance of the SHM framework. Finally, the conclusions end the paper.

## 2. From Data Acquisition to Classification

**Figure 1:**
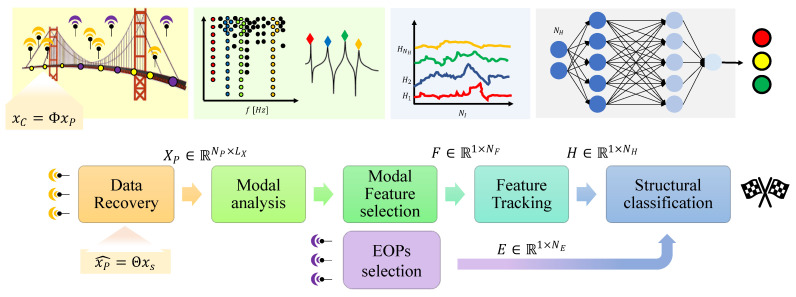
Proposed framework for structural assessment: From left to the right, data compression and recovery, modal feature extraction and selection with the final structural assessment block. The matrix $X_{P}$ is used to indicate the ensemble of CS-reconstructed signals from all the different acquisition points, as it is required by OMA algorithms to provide a global understanding of the structure under analysis.

The monitoring framework proposed in this work (summarized in Figure 1) is organized around three successive steps: (i) the *data compression and recovery* phase, which is aimed at retrieving the original time waveform from compressive acquisitions; (ii) the *modal identification* procedure, returning the structural features of interests; and (iii) the final *classification* stage, which leverages ML techniques as enabling tools for structural integrity assessment.

Moreover, for each of these phases, an efficient processing strategy to deal with it was included. To this end, it is worth stressing the fact that the selected algorithms only represent some of the many possible solutions, and their selection has to be properly judged depending on the characteristic of the scenario under test and the available instrumentation.

### 2.1. Data Compression and Recovery

This processing phase includes data cleansing (such as trend removal and filtering) and compression procedures that are performed by local sensor nodes installed on the structure [40]. Subsequently, compressed data are transmitted to a central aggregating unit where the original time series is recovered.

In CS-based strategies, the compression procedure can be modelled by the product $x_{C}=\Phi x_{P}$, where $x_{P}$ is the generic signal acquired by one of the $L_{x}$ peripheral sensors, $\Phi \in R^{N_{C}\times N_{P}}$ is the rectangular *sensing* matrix ($N_{C}\ll N_{P}$) and $x_{C}$ is the compressed signal, which is eventually forwarded by the peripheral node to the central processing unit. Here, a *recovery procedure* is applied for each of the active sensors, which consists in estimating ${\hat{x}}_{P}$ from the assumption that such signal is sparse in a representation domain spanned by a given basis $\Theta \in R^{N_{P}\times Q}$, i.e., $x_{P}=\Theta \;\cdot \;x_{S}$, where most of the coefficients in $x_{S}$ are zero valued or negligible. In this general framework, a wide range of variants were investigated by selecting different sensing matrices, different representation bases and/or different optimization procedures for the recovery of the sparse coefficients [41].

Among the different strategies implementing CS, the model-assisted rakeness-based compressed sensing technique (MRAK-CS) [19] is suggested in this work for data compression and recovery thanks to its peculiar adaptation to the second order statistics, i.e., to the signal energy distribution, of the processed data. This is extremely beneficial for vibration analysis, where the structural properties are defined in the spectral (frequency) domain. Compared with alternative CS approaches, MRAK-CS not only exploits the classical sparsity assumption, but it specifically leverages the available prior information about the structure, namely the fact that the energy is not uniformly distributed over the whole spectrum but rather concentrated near a few spectral components, as a ruling criterion for the optimization of the sensing matrix.

**Figure 2:**
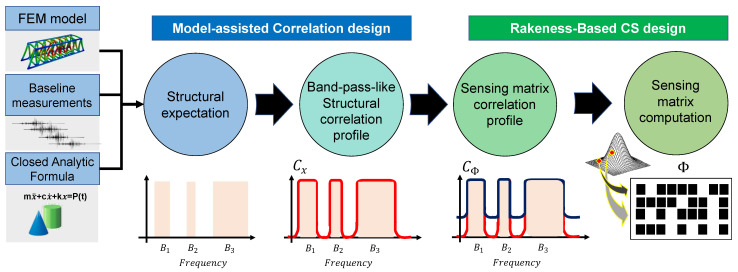
General processing flow at the basis of the MRAK-CS approach.

The strategy to be implemented for the derivation of the optimal sensing matrix as dictated by the MRAK-CS approach is schematically represented in Figure 2. The computation starts with the selection of the frequency regions of interest, as they can be predicted by a numerical model or prior structural campaigns, on the basis of which a band-pass-like correlation profile of the structure ($C_{x}$) is synthetically designed. Then, the CS-based problem statement is entered by firstly extracting the sensing matrix correlation profile ($C_{S}$) as prescribed by the analytical solution of rakeness-based approach, i.e., $C_{S}=\frac{1}{N_{C}}\left(\frac{C_{X}}{\mathrm{tr}(C_{X})}+\frac{I_{N_{C}}}{N_{C}}\right)$ (in which tr(·) stands for the matrix trace operator, while $I_{N_{C}}$ is an $N_{C}\times N_{C}$ identity matrix). Hence, the latter value is used to sample each row of the sensing matrix Φ from a multivariate Gaussian distribution with zero mean and correlation profile equal to $C_{S}$.

### 2.2. Modal Parameter Extraction

Modal identification can be performed in the time or frequency domain. The selection of one category over the other is usually influenced by the required frequency resolution, which is a consequence of mode proximity and the allowable computational complexity [14]. The basic idea of frequency-domain strategies is to derive modal parameters from quantities (i.e., magnitude, phase, half-bandwidth) associated with the peak values of the spectral response function [42].

Conversely, stochastic subspace identification (SSI) algorithms [43] tackle the problem from a time-domain perspective, by modelling the acquired time series as the output of an equivalent linear system whose governing equations are completely described by the related state–space matrices. The principal advantage of SSI methods over conventional spectral alternatives relies in their fully unsupervised nature. Indeed, the extraction of modal features can be automated and used as input for AI tools. For this reason, it constitutes the core block of the modal identification task considered in this work.

The crucial point of SSI methodologies is the selection of the most appropriate model order, on the basis of which the system matrices are computed and the corresponding modal parameters can be retrieved by means of an eigenvalue decomposition. It is worth noting that the model order depends on the operational and environmental conditions, therefore such a parameter must be adaptively selected. To tackle this issue, the so-called stabilisation diagram [43], i.e., a point chart representing how the location of the identified modal frequency values may vary as a function of increasing order number, can be employed. When paired with clustering procedures, this tool provides valuable information about the frequency vector *F* containing the $N_{F}$ identified structural modes, in a totally unsupervised manner. Once modal frequencies were estimated with the clustering procedure, their evolution over time can be tracked with Gaussian moving average filters which aim at fitting a Gaussian kernel function to each *h*-th vibration component of interest: At each step, the filter is designed to update the mean value $\mu_{h},h,1\ldots N_{H}$ with the average value of the frequency points falling in a frequency interval of ±2σ, σ being the standard deviation. This further step is essential for three main motivations: (i) Keeping trace of slow variations induced by environmental effects, (ii) filtering out spurious components which are not consistent across successive measurements and (iii) shrinking the dimensions of the feature space to $N_{H}\leq N_{F}$ components of interests.

#### A Covariance-Based SSI Approach

**Figure 3:**
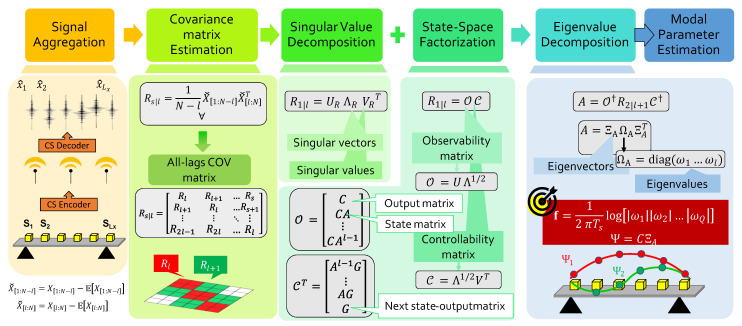
Schematic representation of the SSI-COV processing flow, from data collection to modal parameter extraction.

Amidst the various SSI implementations, the SSI-COV method in Figure 3 deserves particular attention owing to its robust recovery without affecting the processing time. SSI-COV takes its name from the calculation of the covariance function of the measured data, that represents the core function of the entire algorithm. Given this defining feature, it is worth observing that SSI-COV represents an optimal complement to the MRAK-CS compression approach described beforehand: This due to the fact that both strategies, even if tackling different aspects of the monitoring chain, are covariance-based, i.e., they both aim at extracting structural information from the covariance function of vibration signals while maximizing the preserved energy. For this reason, when combined with compression techniques, SSI-COV could be more advantageous with respect to other feature extraction strategies since it is implicitly less prone to possible signal reconstruction errors at the end of the CS recovery phase. It exploits the concept of state variables for casting the driving structural equations into a mathematical system of *Q* first-order differential equations that emulate the dynamics of the underlying physical (structural) system.

The approach involves the following steps:

1. Compute, for fixed time lag *l* and time shift *s*, the block Toeplitz matrix of dimension $L_{x}l\times L_{x}l$
  (1) $$R_{s|l}=\left[\begin{array}{cccc} R_{l} & R_{l+1} & \cdots & R_{s}\\ R_{l+1} & R_{l} & \cdots & R_{s+1}\\ \vdots & \vdots & \ddots \vdots \\ R_{2l-1} & R_{2l} & \cdots & R_{l} \end{array}\right]$$
  in which the internal $L_{x}\times L_{x}$ blocks:
  (2) $$R_{l}=\frac{1}{N-l}\left[X_{\left[1:N-l\right]}-E\left[X_{\left[1:N-l\right]}\right]\right]{\left[X_{\left[l:N\right]}-E\left[X_{\left[l:N\right]}\right]\right]}^{T}\in R^{L_{x}\times L_{x}}$$
  are nothing but the covariance matrix between the aggregated output signals $X\;=\;[x1\ldots x_{L_{x}}]$ acquired in the interval [1:N−l] and [l:N], respectively.
2. Perform the Singular Value Decomposition (SVD) of $R_{1|l}$ (s=1), returning $R_{1|l}=U_{R}\Lambda_{R}V_{R}^{H}$, with $U_{R}\in R^{L_{x}l\times Q}$ the rectangular matrix of left singular vectors and $\Lambda_{R}\in R^{Q\times Q}$ the diagonal matrix of singular values.
3. Apply the state–space factorization of the covariance matrix. Starting from the pure algebraic manipulation of the SVD, one may write $R_{1|l}\;=\;U_{R}\Lambda_{R}V_{R}^{H}=OC$. This means that $R_{1|l}$ can be decomposed into the product of two matrices: The so-called observability matrix $O=U\Lambda^{1/2}$ and the controllability matrix $C=\Lambda^{1/2}V^{T}$. The advantage in pursuing such factorization is that the two latter quantities admit an alternative state–space formulation as $O^{T}=\left[\begin{array}{cccc} C & CA & \ldots & CA^{l-1} \end{array}\right]$ and $C=\left[\begin{array}{cccc} A^{l-1}G & \ldots & AG & G \end{array}\right]$ uniquely determined by the state output matrix *A*, the state matrix*C* and the next state–output matrix *G*. While *C* and *G* can be easily extracted from the first *Q* rows (columns) of the controllability and observability matrix, respectively, the computation of *A* is given by $A=O^{†}R_{2|l+1}C^{†}$ (${}^{†}$ being the Moore–Penrose pseudoinverse operator).
4. Execute the eigenvalue decomposition of the above-computed state matrix. This is decomposed as $A=\Xi_{A}\Omega_{A}\Xi_{A}^{T}$, corresponding to the product of the eigenvector matrix $\Xi_{A}\;\in \;R^{Q\times Q}$ and the diagonal matrix of *Q* eigenvalues $\omega_{q}$, namely $\Omega_{A}=\mathrm{diag}\left[\omega_{1},\ldots ,\omega_{Q}\right]$.
5. Estimate the sought natural frequencies of vibration *f* and mode shapes Ψ from ($T_{s}$ being the sampling time):
  (3a) $$\begin{array}{ccc} f & = & \frac{\left|\log(\mathrm{diag}\left(\Omega_{A}\right))\right|}{2\pi T_{s}} \end{array}$$
  (3b) $$\begin{array}{ccc} \Psi & = & C\Xi_{A} \end{array}$$

As a competitive variant to SSI-COV, in SSI-DATA the computation of the covariance matrix is replaced by the projection of the row space of future outputs into the row space of past outputs. The problem with this method is that it involves the factorization of a very large matrix and, hence, becomes very computationally onerous. For this reason, SSI-COV inherently provides a much faster and efficient algorithmic solution, since the derivation of $R_{s|l}$ can easily be obtained via the Fourier transform. This is also one of the reason why SSI-COV is preferred over SSI-DATA in decentralized monitoring systems where near-sensor data processing and feature extraction is a trending research direction [44]. Beside, alternative solutions such as the contemporary canonical correlation analysis [45] have very recently been proposed for real-time structural analysis, showing the superior capability of providing a more robust identification method which is less sensitive to EOP uncertainties.

### 2.3. Environmental Analysis

Modal parameters are extremely sensitive to environmental factors (e.g., temperature and humidity) since they are constitutive elements determining the stiffness and damping property of the structure [13].

To cope with environmental factors, the conventional approach is to resort to regression methods [28]. Conversely, the approach presented in this work tackles this problem from a pure ML perspective, by including $N_{E}$ EOP parameters (grouped into the $N_{E}$-dimensional vector *E*) as additional input features of the AI block. In this manner, the neural network is instructed to autonomously learn this frequency vs EOP relationship, without requiring any further processing steps to be performed aside.

### 2.4. Neural Network Design

OCCs can be seen as standard neural networks trained with samples acquired for the pristine structure since no training data is usually available for damaged conditions (i.e., the so called *adversarial population*). In these cases, a possible alternative consists in artificially generating these adversarial points. Among the OCC implementations presented in the literature, the very recent OCCNN proposed in [46] and the Autoassociative Neural Network (ANN) are considered in this work.

#### 2.4.1. OCCNN

**Figure 4:**
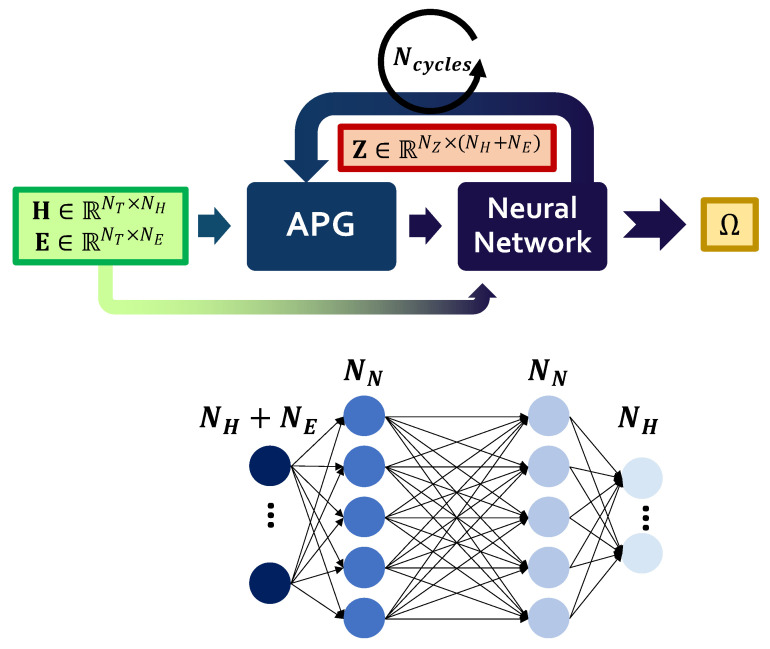
General scheme of OCCNN with its main procedural blocks.

OCCNN [36] is an ML technique aiming at finding either linear or non-linear boundaries between healthy and defective conditions in the parameter space. In these terms, it serves the goal of anomaly detection in low feature space by predicting whether an input feature vector falls into a normal or abnormal region of the total feature space. Let us suppose that $N_{T}$ measurements are used in the training phase, whose corresponding structural and EOP features can be organized in the matrices $H\in R^{N_{T}\times N_{H}}$ and $E\in R^{N_{T}\times N_{E}}$, respectively. As schematically drawn in Figure 4, the neural network topology consists of two main elements. The former is the adversarial point generator block (APG), which randomly generates data $Z\in R^{N_{Z}\times \left(N_{H}+N_{E}\right)}$ of the defective class in the damaged space identified in a given iteration, where $N_{Z}$ indicates the number of adversarial points to be used during training. As described in [46], APG generates points in an iterative way by sampling them from a uniform random distribution, assuming their distribution in the feature space can be described as a Poisson point process with density $\lambda_{i}$ (if *i* is the current iteration index). The latter value can be computed as [47] $\lambda_{i}=\frac{\sum_{n=1}^{N_{T}}k_{n}-1}{\pi \sum_{n=1}^{N_{T}}r_{n}^{2}}$, in which $r_{n}$ indicates the Euclidean distance between the *n*-th point and its $k_{n}$-th neighbour and $N_{T}$ is the number of training points. More formally, at each step, the APG block takes as inputs the point density and the weights of the NN that represent the network state after the previous iteration and defines the actually estimated boundaries Ω ($\Omega_{0}$ being the null matrix so that points can be generated in the whole space): The adversarial points are thus generated in the portion of the feature space where the training points are absent and the output layer activation function is greater than 0.5 (assuming that label 1 is associated with the training set points and 0 to the adversarial ones) [46].

These adversarial data, together with healthy instances, are plugged as inputs to the second NN component, that is a two-layer fully connected NN with $N_{N}$ neurons in each hidden layer, finally providing an estimate of the boundaries Ω.

The adversarial point generator cycles are iterated until the desired level of fitting with respect to the training data distribution is reached. The higher the number of cycles, the higher the resolution of the boundary contours will become. It follows that two key variables might significantly affect the classification performance of OCCNN, which are the number $N_{cycles}$ of APG iterations retained sufficient for a robust system realization, and the number of neurons per layer.

#### 2.4.2. Autoassociative Neural Network

**Figure 5:**
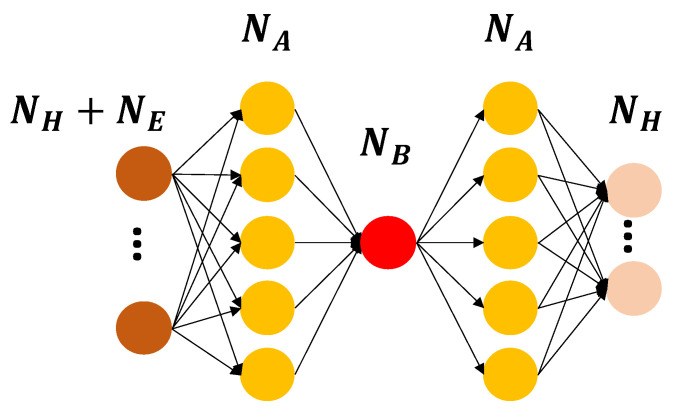
General scheme of the implemented ANN architecture.

In essence, an ANN [48] (Figure 5) represents a feed forward multilayer NN whose goal is to reconstruct data as they appear at the input layer (a condition which implies an identical number of neurons in the input and output layer). The processing chain involves a compression stage, in which the dimensions are reduced by means of a mapping function with progressively lower neurons per layer, followed by a reconstruction step, also known as demapping layer. The role of the mapping layer (with $N_{A}$ neurons) is to project the input data into a lower dimensional space that is used as a bottleneck layer thanks to a number of neurons $N_{B}$ lesser than the dimensions of the feature space; an opposite function is conversely fulfilled by the demapping counterpart. Anomaly detection is achieved by searching for abrupt variations in the residual (i.e., reconstruction error) between the input and the currently predicted output values.

## 3. Experimental Validation

### 3.1. Z24-Bridge Dataset

The openly available dataset related to the Z24 bridge [37] provides unique features for the assessment of SHM algorithms. The Z24 bridge was monitored for more than one year by means of a permanently installed monitoring network consisting of 11 uni-axial accelerometers and multiple environmental sensors (humidity, temperature, wind). Two different experimental campaigns were performed: A long term continuous test, during which the structure was subjected to operational excitation, and a progressive damage test, consisting of purposely induced deterioration processes. The monitoring system was programmed to acquire, on an hourly basis and from all the installed accelerometers, 65,536 acceleration values at a sample rate of 100 Hz, corresponding to observation windows of 11 min. Unfortunately, some measurements were lost due to sensor failures, such that only 55.6% of the total data are now available. Among the total $N_{I}=5651$ observations, 4922 instances belong to the normal class which is acquired in healthy structural conditions, while the remaining 729 instances are acquired in damaged configurations.

When compared with previous works related to the same dataset, the analysis presented here is novel in that it jointly evaluates three pivotal aspects for the design of the next generation of SHM architectures, which are: (i) The investigation of the effects of adapted compressed sensing on the quality of the identified bridge health status, (ii) the exploitation of temperature data as direct input of the NN models, (iii) the inclusion of instrumental non-idealities on the entire monitoring processing flow, and in particular of the residual noise density characterizing commercial off-the-shelf MEMS accelerometers.

A dataset preparation phase was necessary, too. In particular, the machine learning models adopted in this work only need data from the normal class during training. Thus, 70% of these data from the normal class was randomly sampled to favor diversity in terms of environmental conditions. This subset was further subdivided: 70% of it was used for training and the leftover 30% allocated to validation. Conversely, the remaining 30% of the normal class together with all the data acquired in damaged conditions were employed for testing purposes.

### 3.2. Data Compression and Recovery

For the target scenario explored in this work, modal analysis studies applied to the Z24 dataset proved that the most relevant modal components of the bridge are located below 20 Hz. In particular, at a reference temperature value of 25 °C and in nominal working conditions (no vehicle passing through), the three dominant bending modes are located at 3.87 Hz, 12.42 Hz and 13.21 Hz, one lateral mode at 4.82 Hz and two closely spaced mixed torsion/bending modes at 9.77 Hz and 10.50 Hz, respectively. By taking into consideration these modal frequencies, two main spectral bands of interest were identified and plugged as input of the MRAK-CS approach, used for the estimation of the compression matrix: The former one spans the interval [3.5;5] Hz, while the latter has wider dimensions and includes all the components from 9.5 Hz to 13.5 Hz. This information was used to design the sensing matrix, as illustrated in [19], while the Discrete Cosine Transform (DCT) matrix was assumed as sparsifying basis Θ.

The acquired waveforms were divided into 512-sample long segments and the compression ratio, intended as the ratio between the number of columns ($N_{P}$) and rows ($N_{C}$) of the sensing matrix, was set equal to 6. These parameters for the CS encoder were selected to be compatible with real-field scenarios where the storage capabilities of edge devices are limited to a few hundred kB. Indeed, assuming each piece of data can be represented as a 4 B word, storing a sensing matrix with the selected dimensions (i.e., 512/6=85×512 elements) might require at least 90 kB. It is worth emphasizing that the imposed compression level is higher than typical values adopted in vibration analysis [19,49]; in these terms, it was chosen to replicate worst case scenarios. Finally, the SPGL1 [50] algorithm was employed for the recovery of the sparse coefficients. The underpinning principle behind SPGL1 is that the recovery process can be treated as a convex optimization problem aiming at estimating the set of sparse coefficients ${\hat{x}}_{P}$ of the original signal such that the Euclidean norm of the error with which the received compressed signal $x_{C}$ is matched by the currently predicted solution ${\hat{x}}_{C}=\Theta \Phi {\hat{x}}_{P}$ is minimum. Compared with different solvers proposed in the literature, the convex-based nature of SPGL1 allows this method to achieve better reconstruction accuracy since it does not suffer from approximation errors characterizing greedy or iterative algorithms [51].

### 3.3. Feature Extraction

#### 3.3.1. Modal Identification

As anticipated, the covariance-based SSI-COV approach in Figure 3 was adopted to estimate the main vibration components of the bridge. More specifically, the structural identification process was divided into three steps. Firstly, the stabilisation diagram was computed for a model order ranging from 1 to $N_{F}=160$; then, the *k*-means algorithm [52] was run to create a batch of candidate modal frequencies. To this end, the number of centroids for each instance was varied from 10 to 15 and the corresponding Euclidean distance (intended as the sum of the distances between the centroids and the associated points) was computed. The number of centroids returning the lowest error has thus been chosen for the given set of measurements. The adoption of such a blind approach is due to the fact that the actual number of frequencies in each data record is not known a priori and needs to be adaptively estimated. Thirdly, only the first bending and lateral modes were retained in the following analysis (i.e., $N_{H}=2$) due to their high energy content and, consequently, more pronounced response at the occurrence of structural anomalies. The moving average filter was finally applied to track their evolution over subsequent instances. In this case, the parameters of the kernel Gaussian functions for the first and second modal components were selected to be $\mu_{1}=4.0\mathrm{Hz}$ and $\mu_{2}=5.2\mathrm{Hz}$, respectively, while an equal standard deviation of 0.16 Hz was imposed to ensure the best compromise between the capability of the filter to react to rapid frequency changes while being immune to potential outliers.

#### 3.3.2. EOP Selection

The environmental monitoring system deployed on the bridge consisted mostly of temperature and humidity sensors, which were deployed in a redundant configuration (more than 53 different measurement positions) over the whole structure, so as to precisely keep trace of EOP effects on the vibration signature. As already proven in previous works for the Z24 use-case [53], very high correlation was found between the frequency shifts induced by thermal excursion and the temperature variation at the top deck of the structure. Thus, such quantity was the only one retained for the subsequent classification.

**Figure 6:**
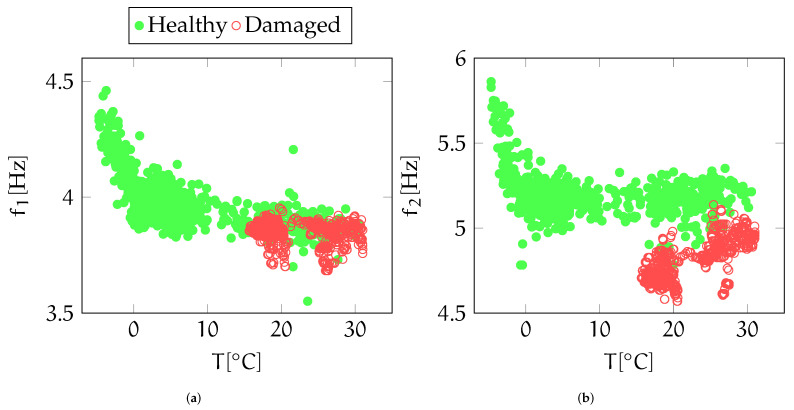
First (**a**) and second (**b**) frequency component vs temperature. Red points concentrating on high temperature values (from 20 °C to 30 °C) are related to damaged structural configurations, while the green ones covering the entire temperature axis refer to the healthy state.

It is worth underlining that, thanks to the relatively high thermal inertia of the structure, just one temperature value per acceleration series has to be stored. Trends in modal frequencies induced by temperature fluctuations are depicted in Figure 6 for the first (Figure 6a) and second (Figure 6b) vibration component.

### 3.4. Neural Network Models

The details of the explored NN models are presented in this subsection. For the sake of brevity and from this point onward, the acronyms OCCNN and ANN will be denoted as *O* and *A*. The initial number of parameters for the NN models was selected to provide a fair comparison with the reference work in [36,46], in which the OCCNN architecture was firstly proposed. it is noteworthy that such a choice was necessary to evaluate how, with the remaining parameters being equal, the effect of temperature data could actually impact on the quality of the classification performance. Then, starting from this initial configuration, the model complexity was progressively reduced to shrink the inference time and make the solution compatible with edge devices. Coherently, the basic OCCNN implementation, as it was proposed in [46] consisting of two hidden layers with $N_{N}=50$ neurons each, is referred to as model A ($O_{A}$); conversely, subscripts ${}_{B}$, ${}_{C}$ and ${}_{D}$ will be used to indicate its distilled versions with 32, 16 and eight neurons per layer, respectively. Besides, the number of adversarial points was generated as detailed in [46].

For the ANN case, $N_{A}=64$ neurons were selected in the mapping and demapping layer, a quantity which was determined by taking into consideration the complexity of the problem at hand with respect to the available number of instances used during training, whereas one single neuron was used for the bottleneck layer, as constrained by the exploitation of only two features in the input stage.

When the networks are fed with CS data, they will be indicated with superscript ${}^{CS}$, while the ones complemented with temperature values are named after with prefix *T* (e.g., $TO_{A}$, $TA_{1}$). Rectified Linear Unit (ReLU) was chosen as activation function for the input and hidden layers, while softmax was considered in the output layer for all the investigated NN models. The number of training epochs was set equal to 5000 with a learning rate of the stochastic gradient descent equal to 0.05. Cross-validation with k-fold=5 was also considered to avoid biases in the designed classification models.

### 3.5. Noise Density in MEMS Accelerometers

Micro Electro-Mechanical Systems (MEMS) devices are characterized by high sensitivity, low-power consumption and very high integration levels, which made this sensing technology a cost-effective yet reliable and extremely advantageous alternative to the piezolectric counterpart for the design of accelerometer sensors [54]. Indeed, the advent of MEMS sensors made the widespread development of low-cost, dense and miniaturized sensor networks a real opportunity in the context of SHM [55].

Notwithstanding their successful adoption, signals acquired by MEMS accelerometers are affected by comparatively higher intrinsic noise density values, which thus need to be properly accounted for in the signal processing chain to assess the actual performances of the implemented algorithms. To this end, further analyses were performed in this work, in which the original data were degraded by adding the residual noise floor inherent in two different kinds of digital accelerometers. The noise was generated via the Matlab^®^ Sensor Fusion and Tracking Toolbox™, which offers specific routines to simulate the mechanical behavior of inertial measurement units, including accelerometers and gyroscopes.

In more detail, the mechanical characteristics in Table 1 were assumed: As can be noticed, MEMS accelerometer type MA ($N_{o}=25\;\mu g/\sqrt{\mathrm{Hz}}$) refers to commercial off-the-shelf devices exhibiting the lowest noise density levels, while type MB with $N_{o}=80\;\mu g/\sqrt{\mathrm{Hz}}$ is representative of medium-class but extremely low-cost devices. Coherently, the newly obtained waveforms were then processed following the procedures exposed in Section 3.2 and Section 3.3 while maintaining unaltered all the remaining parameters.

For the sake of NN validation, noise-corrupted data can be treated as novel datasets and, for this reason, they are taken as inputs to the previously trained $TO_{D}$ model (the one with noise-free data). This verification procedure was preferred over the generation of new models for each of the new datasets since it represents a more severe test to be passed. At the same time, it is also appropriate in view of practical implementations, in which the variability and the uncertainties hidden in the acquired data cannot be predicted *a priori*.

## 4. Results

Four main objectives were pursued within the experimental validation phase: (*i*) Assess the improvement brought by the introduction of temperature values as additional input features of the AI block; (*ii*) evaluate the effect of compression/recovery stages on the classification performance of the designed SHM framework; (*iii*) reduce the complexity (i.e., number of parameters) of the NN models to be compatible with embedded processors without impinging on the accuracy of the classification; (*iv*) evaluate the effect of MEMS noise floor on classification performances to cope with real issues.

To quantify the performance of the classifiers, four classical classification metrics [1], i.e., accuracy, precision, F1 and recall, were computed.

### 4.1. Effect of Temperature Data

**Figure 7:**
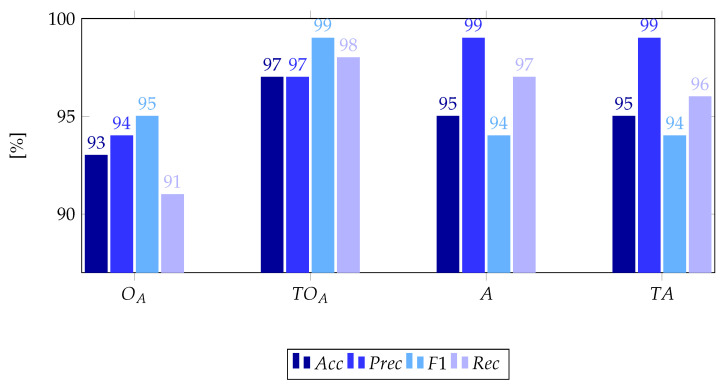
Performances of OCCNN and ANN reference models with (TO,TA) and without ($O_{A}$,$A_{1}$) temperature input values, for compression-free configurations.

As can be observed in the bar chart depicted in Figure 7, adding temperature values as additional input features to the NNs provides invaluable insight in the case of the the OCCNN solution, for which an average increase of 4.5 percentage points was observed while moving from the basic $O_{A}$ model to the $TO_{A}$ one corrected with temperature data. Conversely, in the ANN implementation, no consistent gain in the quality of the classification process is obtained by inputting temperatures. A possible explanation is in the compression imposed by the bottleneck layer, which acts as a filtering operator removing noise and minor details from the input signals [56]. This condition also applies to the framework analysed in this work, where the detection of structural anomalies is performed on the reconstructed frequency features at the output layer while disregarding the additional temperature data used in the input stage. Based on these observations, only temperature-added OCCNN realizations will be investigated hereinafter.

Moreover, it is important to point out that classification scores attained by $TO_{A}$ are highly competitive to the ones presented in [36], which explore a combined ANN-OCCNN architecture to account for biases in the training point density estimation. In the ANN-OCCNN case, an ANN is employed in the first step of the classification chain and used to generate adversarial points rather than resorting to a first cycle of OCCNN to derive a rough estimation of the feature boundaries, while totally neglecting the exogenous contribution of EOPs on modal features. Despite its remarkable accuracy (96% accuracy and 98% precision), the ANN-OCCNN solution is poorly compatible with the inclusion of temperature values and CS compression/decompression stages due to the filtering effect at the basis of ANN.

When the current results are compared with those presented in [39], where PCA is employed to decouple the impact of EOPs and structural damages, the $TO_{A}$ solution here proposed performs satisfactorily well, allowing to discern between healthy and deficient configurations in a completely agnostic way, without needing to find, among all the principal components yielded by PCA, the one which better allows to decouple the effect of temperature variation from true damage. Indeed, as demonstrated in [39], some principal components might be completely blind with respect to the existence of damage and, thus, may lead to an erroneous structural bulletin if not aided by a purposely dedicated training phase in which the sensitivity of this principal components is firstly assessed to select the best indicator.

### 4.2. Effect of Data Compression

**Figure 8:**
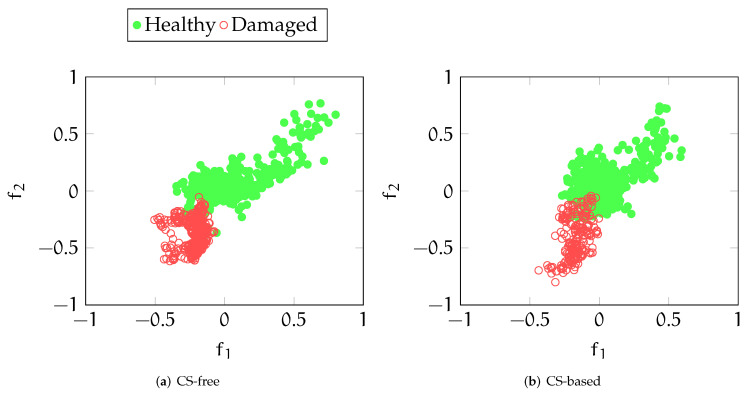
Feature space distribution with compression-free (**a**) and CS-processed (**b**) processing framework. Red points distributed in the south-west part of the feature space are referred to the damaged state, while the green ones represent healthy structural configurations.

The primary impact of compression/decompression stages can be observed in the larger superposition between healthy and damaged data in the feature space distribution depicted in Figure 8. Coherently, a reduction in the performance of the CS-driven versions can be seen in the first column of Table 2 for OCCNN ($TO_{A}^{CS}$) with respect to the results illustrated in the previous section. Similarly as before, the inclusion of temperature in the pool of NN inputs is particularly effective even in this case since it returns classification results comparable to those pertaining to the basic $O_{A}$ alternative. Indeed, despite a minor reduction in the precision, the accuracy is almost equivalent and F1 and recall undergo a significant improvement.

### 4.3. Effect of NN Distillation

Finally, the computational cost, here intended as the number of NN parameters, was reduced to make the NN model compatible with the constrained resources of embedded devices, and the effect on the classification accuracy was thus evaluated. To this end, starting from the network with $N_{N,A}=50$, the number of neurons per layer in the OCCNN architecture was then reduced to 32, 16 and down to 8, corresponding to a shrinkage of model parameters to $N_{N,D}=122$, with intermediate values of $N_{N,B}=1250$ and $N_{N,C}=370$. In the first three lines of Table 2, other performance metrics, such as the memory usage and the execution time of each distilled version, are enclosed to provide a more accurate comparative analysis. Besides, classification scores are also included.

The exponential decrease in both the occupied memory and running time of the algorithms as $N_{N}$ halves can be clearly observed, the combined action of which leads, in turn, to a consistent contraction of the associated power consumption. Indeed, the model size shrinks more than 95% with a time gain above 75% while $N_{N}$ moves from 50 to 8 neurons.

**Figure 9:**
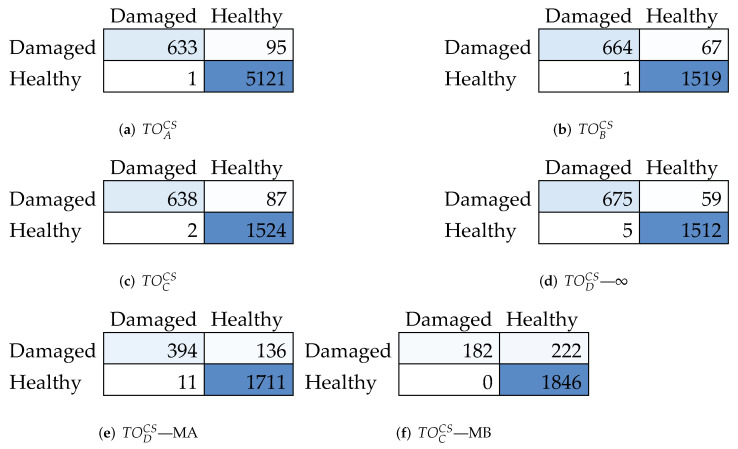
Confusion matrices corresponding to the classification scores presented in Table 2.

Remarkably, model *D* with only eight neurons attains high classification performances, which are absolutely competitive with the ones associated with the most redundant configuration (model *A*). Moreover, it performs even better than alternative solutions with much higher parameters (see models *B* and *C*). This is further justified by the very similar classified instances reported in the corresponding confusion matrices of Figure 9a–d.

### 4.4. Effect of Intrinsic Noise Density in MEMS Accelerometers

The impact of non-negligible noise floors in MEMS accelerometers can be quantified by observing the last three columns in Table 2 (header MA and MB). As can be observed, the primary effect is the decrease up to seven percentage points of the accuracy in the classified instances while moving from noise free (header *∞*) to MB MEMS-type, a trend which is also clarified by the confusion matrices (e) and (f) in Figure 9. A similar trend is evidenced for precision and F1; however, good performance values are attained, which are always very close or consistently above 90%. Recall demonstrates to be less sensitive to such noise levels, being nearly constant at 100%. On the other hand, MEMS typology MA, which features a less significant $N_{o}$ level, only undergoes a limited reduction in both accuracy and precision (which show losses of 3% and 4%, respectively).

As such, it was demonstrated how the selection of the sensing unit might play a crucial role in the effectiveness of the adopted signal processing techniques, which must cope with the inherent source of non-idealities while moving from theoretical analyses to real case studies.

## 5. Conclusions

In this work, an investigation on how data compression may affect the performance of ML-based damage detection procedures in structural health monitoring is presented. In particular, one of the most promising compression techniques (namely MRAK-CS) was applied to the dataset of the Z24 bridge and the recovered data were used to feed low-complexity neural networks to perform the anomaly detection task.

In particular, it was shown: (i) How the compression ratio influences the detection performances; (ii) the different degradation in the performance achieved with different classifiers (ANN and OCCNN) as a function of the network complexity; (iii) the practical implementation of the model in low-power and resource constrained devices; (iv) the beneficial effect brought by the inclusion of temperature data among the inputs of the network; and (v) the impact of non-negligible noise floors on MEMS-based accelerometers on classification scores.

From the results, it can be concluded that the OCCNN architecture with eight neurons per layer and a compression ratio equal to 6 achieves a negligible degradation with respect to much deeper networks applied to features extracted from uncompressed data. The low computational cost of the implemented network is compatible with the storage and processing resources of low-cost microcontrollers, and the compression stage allows to minimize the risk of network congestion. Further works will include the validation of the proposed approach in multiple application scenarios and the inclusion of additional features, such as damping factors and mode shapes, as inputs for the NNs, in order to further improve the classification results.

## Figures and Tables

**Table 1:** Mechanical features of the considered MEMS accelerometer types.

Feature	Unity of Measure	MA	MB
Sensitivity @ ±2g	μg/LSB	61.0	3.9
Zero-*g* level offset	mg	40	25
Noise ($N_{o})$	$\mu g/\sqrt{\mathrm{Hz}}$	80	25
Zero-*g* change vs temperature	mg/C	±0.1	±0.1
Sensitivity change vs temperature	[%/C]	±0.01	±0.01

**Table 2:** Performance metrics of OCCNN models A, B, C, D with temperature input values and CS-processed configurations: Beside the classical classification scores, the overall complexity, in terms of memory consumption, number of parameters and execution time, is enclosed.

$N_{N}$	${\mathrm{TO}}_{A}^{\mathrm{CS}}$	${\mathrm{TO}}_{B}^{\mathrm{CS}}$	${\mathrm{TO}}_{C}^{\mathrm{CS}}$	${\mathrm{TO}}_{D}^{\mathrm{CS}}$		
				*∞*	MA	MB
Model size [KB]	13.232	6.824	3.304	2.312		
parameters	2852	1250	370	122		
Accuracy [%]	95.73	96.98	96.04	97.16	93.49	90.12
Precision [%]	94.12	95.78	94.59	96.25	92.65	89.25
Recall [%]	99.93	99.93	99.87	99.67	99.37	100
F1 [%]	96.94	97.81	97.16	97.93	96.18	94.04

## Data Availability

Not applicable.

## Abbreviations

The following abbreviations are used in this manuscript:

AI	Artificial Intelligence
ANN	Autoassociative Neural Network
CS	Compressed Sensing
DCT	Discrete Cosine Transform
OCC	One-Class Classifier
OCCNN	One-Class Classifier Neural Network
EOP	Environmental and Operational Parameters
MRAK-CS	Model-assisted Rakeness-based Compressed Sensing
SHM	Structural Health Monitoring
